# Syphilis, the Great Imitator—Clinical and Dermoscopic Features of a Rare Presentation of Secondary Syphilis

**DOI:** 10.3390/ijerph20021339

**Published:** 2023-01-11

**Authors:** Carmen Cantisani, Federica Rega, Luca Ambrosio, Teresa Grieco, Norbert Kiss, Fanni Adél Meznerics, András Bánvölgyi, Giordano Vespasiani, Francesca Arienzo, Giovanni Rossi, Giuseppe Soda, Giovanni Pellacani

**Affiliations:** 1 Dermatology Unit, Department of Clinical Internal Anesthesiologic Cardiovascular Sciences, “Sapienza Medical School” University of Rome, 00185 Rome, Italy; 2 Department of Dermatology, Venereoslogy and Dermatooncology, Semmelweis University, H-1085 Budapest, Hungary; 3 Department of Radiological, Oncological and Pathological Sciences, “Sapienza Medical School” University of Rome, 00161 Rome, Italy

**Keywords:** syphilis, differential diagnosis, dermoscopy, histological finding, insect bites, non-melanoma skin cancer, syphilis

## Abstract

Syphilis is characterized by a wide range of variable clinical symptoms; therefore, it is often referred to as “The Great Imitator”. Here, we report the case of a 69-year-old hepatitis-C-positive MSM patient, who was admitted to our clinic due to a solitary firm painless erythematous maculopapular lesion with a central crater-like crust on the upper right thigh that occurred two months prior. The dermoscopy showed an erythematous, copper-colored, oval lesion with diffuse monomorphic dotted and glomerular vessels, central crust, and circular scaling (Biett’s sign). The histological findings ruled out neoplasia and described a plasma cell infiltrate and endothelial swelling. Finally, the combination of the dermoscopic image, histological findings and the additionally acquired knowledge about the sexual history of the patient at the second visit led to the diagnosis, which was then confirmed with serological tests. Dermoscopy may become a supportive tool to facilitate the recognition of secondary syphilis; however, the reporting of these atypical cases is crucial to highlight the many faces of the disease so that clinicians consider syphilis as part of the differential diagnosis of non-specific lesions.

## 1. Introduction

### 1.1. Sexually Transmitted Infections

Sexually transmitted infections (STIs) are clinical syndromes caused by a wide spectrum of bacteria, viruses and parasites transmitted trough sexual contact [1]. STIs are a global public health issue with increasing prevalence, imposing major economic burdens globally [2,3]. Beside their acute symptoms, they can lead to severe long-term complications, such as pelvic pain, pelvic inflammatory disease, infertility, cervical cancer, arthritis, birth complications and fetal and neonatal damage as a result of vertical transmission [4,5,6,7]. The main factors found to be associated with sexually transmitted infections are unprotected sexual intercourse, promiscuity, past history of STI, present STI infection caused by another pathogen, and receptive anal intercourse [2,8,9,10]. Risk groups with a high prevalence of STIs are adolescents, men who have sex with men (MSM), and transgender individuals [11,12,13].

The prevalence of STIs has shown a significant increase in recent years. Beside socio-economic factors, the progress in the prevention and treatment of Human Immunodeficiency Virus (HIV) can also have a considerable effect on the prevalence of other STIs [14,15]. Although the use of pre-exposure prophylaxis (PrEP) prevents HIV transmission, it also leads to sexual risk behaviors (unprotected sexual intercourse), while not protecting from other STIs. The lockdown during the COVID-19 pandemic highlighted further characteristics of STIs. Several studies found that the incidence of symptomatic STIs remained unchanged regardless of the strict social distancing measures introduced during the first wave of the pandemic, further emphasizing the importance of STI surveillance, as “not having sex is not an option” [16,17,18]. Preventative measures include professional and public education, condom use, and widespread screening [19]. Besides the reimbursement of STI screening, access to point-of-care testing is one of the main gaps in clinical and health services for STI prevention, while telemedicine is a promising but underutilized service for STIs [20]. The emerging use of telemedicine prompted by the COVID-19 pandemic may accelerate the further development of teledermatology systems, establishing the bases of widely accessible patient care worldwide [21,22,23].

### 1.2. Syphilis

#### 1.2.1. Epidemiology

In the United States, syphilis is the third most common bacterial STI after chlamydia and gonorrhea [24]. Risk factors among MSM include methamphetamine use, previous syphilis infection and online dating [25,26,27,28].

#### 1.2.2. Pathogenesis

Syphilis is caused by a facultative anaerobic spirochaete bacterium, Treponema pallidum. Treponemes are highly invasive pathogens that rapidly disseminate after infection [29]. They do not produce endotoxin; however, they cause tissue damage through induced inflammatory processes, with plasma cell infiltration, perivascular inflammation, endothelial cell swelling and proliferation [29]. Due to the lack of surface lipopolysaccharides (LPS) in their cell membrane, a strong innate immune response is not induced by the pathogen, leading to a potentially persisting infection [29,30]. Although the opsonic antibodies cannot neutralize the pathogen effectively, they are highly valuable in the diagnosis of syphilis [31].

#### 1.2.3. Clinical Presentation

The natural history of syphilis is characterized by the cyclical alternation of symptomatic and asymptomatic periods. The first year after infection is the early stage of syphilis, which is divided into primary, secondary, and transient stages. During this phase, the patient is infectious throughout the whole period, even when asymptomatic. The early stage is followed by late or tertiary syphilis, which is less contagious [32].

The primary lesion occurs after a three-week incubation period as a painless ulcerated solitary papule at the inoculation site, while the typical symptoms of secondary syphilis appear 4 to 10 weeks after the exposure [19,29]. The disseminated infection is usually marked by cutaneous generalized lymphadenopathy, which may be accompanied by general malaise and fever [19,33,34,35]. At this stage, the patient is already seropositive [19,31]. Early symptoms of neurosyphilis may also be present and the internal organs can be affected as well [19,33,34,35]. The dominant cutaneous symptom is macular or papulosquamous eruption on the trunk and the extremities, involving palmar and plantar surfaces as well [35]. Confluent nodules of condyloma latum in the genitoanal region or extragenital regions may also be present [35]. Further symptoms include condyloma latum in the genitoanal region or extragenital regions, patchy or diffuse alopecia, and mucosal involvement [35]. Besides the typical manifestations of secondary syphilis, atypical presentations may also occur, including nodular, annular, pustular, framboesiform and nodulo-ulcerative syphilis (lues maligna) [35,36,37,38,39,40,41]. Tertiary syphilis is characterized by granulomatosus reaction, often involving the skin, the cardiovascular and the neurological system [19,29].

#### 1.2.4. Diagnosis

Since Treponema pallidum is virtually non-stained by Gram, special methods, such as darkfield microscopy, immunohistochemistry, silver impregnation, or PCR are required for its detection [19].

Serological tests are currently the best methods for the screening and diagnosis of syphilis. In fact, they are the only available method to detect infection in the latent period, and also help to distinguish between current, untreated infection and previous exposure that has already been treated. Serological tests include non-specific, non-treponemal tests (NTT) and specific or treponemal tests (TT) [42,43]. Non-treponemal tests detect antibodies produced against the antigens released due to tissue necrosis. The most widely used NTTs are the Rapid Plasma Reagin Test (RPR) and Venereal Disease Research Laboratory (VDRL) Test [42,43]. Treponemal tests detect the antibodies produced directly against the pathogen. TTs include fluorescent treponemal antibody absorption (FTA-ABS), microhemagglutination test for antibodies to *T. pallidum* (MHA-TP), *T. pallidum* particle agglutination assay (TPPA), *T. pallidum* enzyme immunoassay (TP-EIA), Chemiluminescence immunoassay (CIA), and *T. pallidum* haemagglutination (TPHA) [42,43,44]. Screening tests include both TTs, NTTs and the combination of the two, while the most widely used tests for disease monitoring are NTTs (RPR or VDRL) [45].

The histopathological features are characterized by signs of immune response against the infection, with superficial and deep perivascular infiltrate containing plasma cells, lichenoid infiltrate obscuring the dermal–epidermal junction, lichenoid as well as superficial and deep perivascular pattern, epidermal hyperplasia, and thickening and/or dilatation of dermal blood [46,47,48,49,50].

#### 1.2.5. Differential Diagnosis

The differential diagnosis of primary syphilis includes several infectious (Herpes simplex virus infection, Staphylococcus aureus infection, chancroid, granuloma inguinale/donovanosis, Lymphogranuloma venereum, vaccinia), and non-infectious diseases (trauma, neoplasm, including squamous cell carcinoma, aphthous ulcer, Behçet disease, fixed drug eruption, zoon balanitis), while secondary syphilis should be differentiated from acute HIV infection, other viral exanthems, pityriasis rosea, drug eruption, psoriasis, erythema multiforme, hand, foot, and mouth disease and Rocky Mountain spotted fever, granuloma annulare, lichen planus, pityriasis rosea, and dermatophyte infection, fungal infection, Kaposi sarcoma, bacillary angiomatosis, foreign body granuloma, lymphoma, lymphomatoid papulosis, pseudolymphoma, leprosy, sarcoidosis and halogenoderma [26,51,52,53].

#### 1.2.6. Treatment

Parenteral Penicillin G is the first line of treatment in every disease stage [54]. In early stage, one dose of 2.4 M units of benzathine penicillin G (BPG) should be administered intramuscularly, while the treatment of late stages requires three doses of BPG (2.4 M units) on days 1, 8 and 15 [45]. Regardless of disease stage, neurosyphilis indicates intravenous drug administration (18–24 million units of Benzyl penicillin daily, for 10–14 days) [45]. Although other agents, such as Azithromycin, Doxycycline or Tetracycline can be used in case of penicillin allergy, some specific settings, including neurosyphilis, tertiary syphilis, syphilis during pregnancy and congenital syphilis, require desensitization to penicillin [45,55].

### 1.3. Dermoscopy

Dermoscopy is a non-invasive diagnostic tool, widely used in the field of dermatology [56]. It allows the in vivo magnification of skin lesions and increases diagnostic accuracy compared to naked-eye examination [56]. The dermoscopic criteria for the differential diagnosis of melanocytic lesions and pigmented and non-pigmented skin tumors are well established, with a wide range of available checklists supporting decision-making in clinical practice [57,58,59]. In contrast, the use of dermoscopy in general dermatology is less common due to the lack of specific criteria and the great dermoscopic expertise of a specialist required for correct diagnosis [60,61,62,63,64,65]. However, dermoscopy can support the diagnosis of several inflammatory diseases, including psoriasis, lichen planus and pityriasis rosea among others, and infectious diseases, such as scabies, common warts, molluscum contagiosum, tick bites or syphilis [60,61,62,63,64,65,66].

#### Dermoscopy of Syphilis

Syphilis has no specific dermoscopic signs or criteria, and there are only a few reported cases focusing on the dermoscopic features of different skin manifestations of secondary syphilis. Erichetti et al. described the palmar lesions of syphilis with an orangish background and a thin, whitish, annular, scaling edge progressing in an outward direction, surrounded by an erythematous halo [67]. They also recorded peripheral telangiectatic vessels, while Mathur et al. described an erythematous maculopapular rash on the forearm and the palm of the patient with scaling and a central darker area fading toward the periphery with an ill-defined border [66,67]. Tognetti et al., highlighted the diffuse monomorphic dotted and glomerular vessels on a diffuse, yellowish-red background of hyperkeratotic palmar lesions, with a circular scaling edge, interpreted as Biett’s sign [53]. Furthermore, a dermoscopic image of erythematous plaques on the penis was characterized by dotted and short linear vessels and peripheral white scaling according to Li et al. [68].

### 1.4. Aim of the Study

Here, we present the challenging case of a 69-year-old man with a single painless erythematous maculopapular lesion to highlight the importance of dermoscopy in general dermatology. In such cases, clinical history and manifestation, as well as non-invasive diagnostic techniques, can help in early diagnosis and prompt treatment.

## 2. Case Report

**Figure 1:**
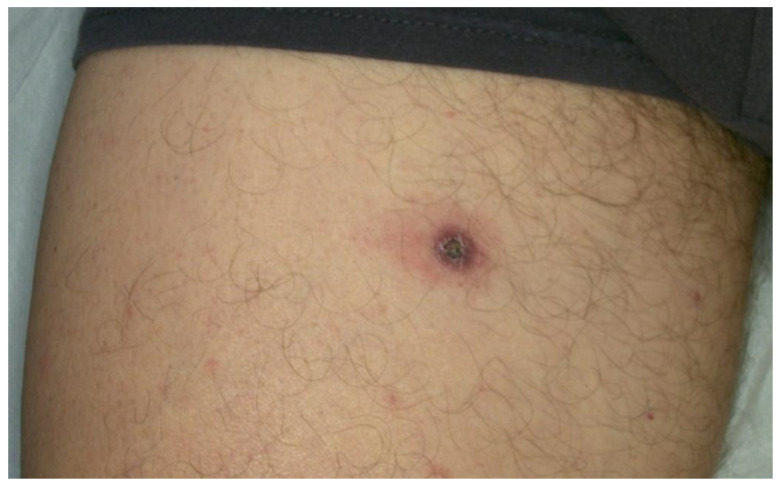
Clinical image of the lesion on the upper thigh of the patient.

A 69-year-old hepatitis-C-positive MSM patient was admitted to the Dermatology Unit of the Department of Clinical Internal Anesthesiologic Cardiovascular Sciences, Sapienza Medical School, University of Rome, Rome, Italy due to a solitary firm painless erythematous maculopapular lesion with a central crater-like crust on the upper right thigh that occurred two months prior (see Figure 1).

**Figure 2:**
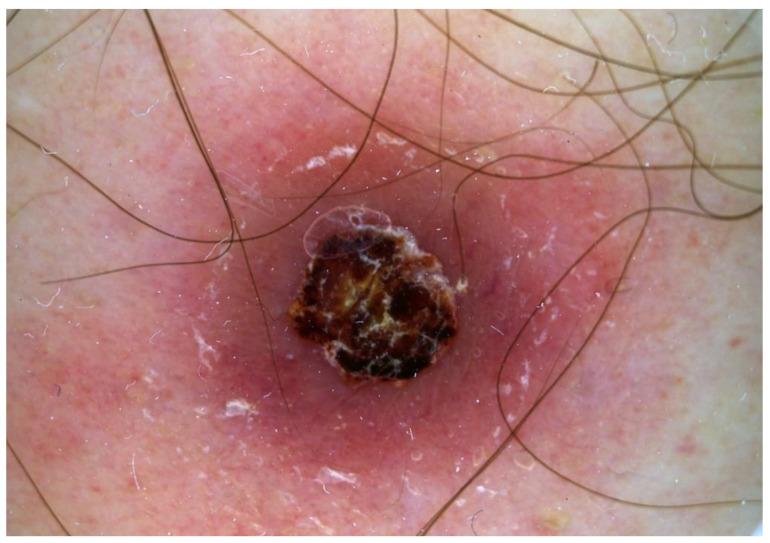
Dermoscopic image showing an erythematous, copper-colored, oval lesion with diffuse monomorphic dotted and glomerular vessels, central crust and circular scaling (Biett’s sign).

The patient described the lesion as enlarging and non-pruritic, and negated associated fever, lethargy, headache, arthralgia, lymphadenopathy, or other novum skin lesion during the first visit. No regional lymphadenopathy, tenderness to palpation, or mucosal involvement was recorded during the physical examination. To exclude non-melanoma skin cancers, a dermoscopic examination and a skin biopsy were performed. The dermoscopy showed an erythematous, copper-colored, oval lesion with diffuse monomorphic dotted and glomerular vessels, central crust and circular scaling (Biett’s sign) (see Figure 2).

**Figure 3:**
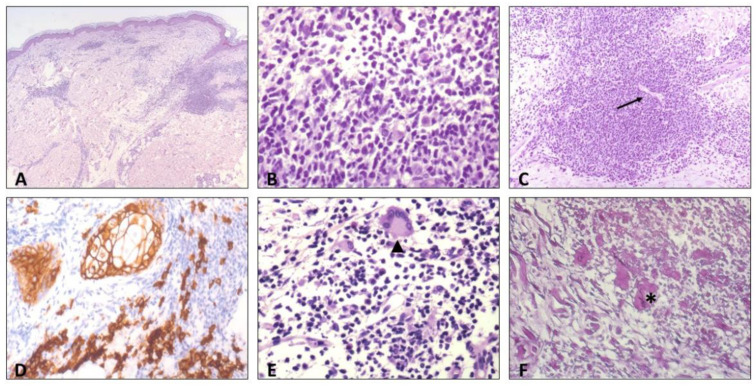
Histologic findings: (**A**) superficial and deep dermal perivascular inflammatory infiltrate, hematoxylin eosin (HE). (**B**) High-power view of the inflammatory infiltrate, showing predominance of lymphocytes and plasma cells, HE. (**C**) Endothelial swelling of dermal blood vessels (arrow), HE. (**D**) Immunostaining for CD138 highlights plasma cells infiltrate, (**E**) scattered multinucleated giant cells in the infiltrate, also of the Langhans type (arrowhead), and (**F**) elastophagocytosis (asterisk), Weigert-Von Gieson staining.

The histopathologic evaluation showed a dermal inflammatory infiltration of lymphocytes, histiocytes, and plasma cells with a superficial and deep perivascular distribution, and the endothelial swelling of dermal blood vessels (Figure 3).

Based on the dermoscopic image, the results of the histological examination and the additional anamnestic data about sexual history and previous syphilis infection provided by the patient during the second visit, the diagnosis of secondary syphilis was established and confirmed by serology (TPPA titer: 1:655,360, Western blot: IgM positive, RPR antibody titer: 1:64). Tests for other bacterial STIs and HIV were negative. After a single dose of 2.4 million UI intramuscular Benzathine Penicillin G, the patient reported the rapid improvement of the eruption.

## 3. Discussion

As syphilis is characterized by a wide range of variable clinical symptoms, it is often referred to as “The Great Imitator”.

Although the diagnosis of syphilis is usually supported by the clinical features, it may be difficult to differentiate it from other annular maculo-papular dermatoses with scaling, especially without information about the patient’s sexual history.

Atypical presentations of secondary syphilis include tinea-like, psoriasiform, impetiginoid, vasculitis-mimicking, lupus-vulgaris-like and lichen-planus-like symptoms, which may occur due to an underlying cause, such as HIV infection [69,70,71,72,73,74,75].

This case report describes an unexpected presentation of secondary syphilis in a 69-year-old man presenting with a solitary lesion in an atypical anatomic area, without the anamnesis of a primary chancre, general symptoms or a known sexual anamnesis indicating syphilis infection.

The symptoms of secondary syphilis are usually systemic due to the hematogenous dissemination of treponemes, with a widely variable lesion morphology and distribution [76]. The presentation of secondary syphilis as a single lesion, or even as localized lesions, is very rare. The localized pattern of secondary syphilis is referred to as the corymbose arrangement, consisting of a greater papule surrounded by smaller satellite lesions [77,78]. However, despite being localized, the lesions of corymbose pattern are multiple. Secondary syphilis presenting as a single lesion is very rarely reported in the literature. Knöpfel et al. reported a rare case of secondary syphilis with a single annular lesion on the scrotum, while Wu et al. described a similar presentation on the jaw [79,80]. In both cases, histology was necessary to establish a final diagnosis, the differential diagnoses including granuloma annulare, tinea, plaque psoriasis, and sub-acute cutaneous lupus erythematosus [79,80]. A solitary lesion of secondary syphilis may also resemble skin tumors, making the diagnosis even more challenging [81]. As our patient presented with a single, mildly erythematous, centrally crusted lesion, with a history of occupational sun exposure, squamous cell carcinoma also had to be ruled out.

In this atypical case, dermoscopy and histology can play an important role in the diagnosis. The dermoscopy showed an erythematous, copper-colored, oval lesion with diffuse monomorphic dotted and glomerular vessels, central crust and circular scaling. The white ring of scaling on the surface of secondary syphilis papules was first described by Laurent-Théodore Biett, now referred to as Biett’s sign [53,66,67]. Although Biett’s sign is a non-specific dermoscopic feature, it might be a useful hint, especially in cases of non-typical syphilis symptoms. Dermoscopy can support differential diagnosis by helping to rule out common cutan neoplasms such as melanoma malignum, basal cell carcinoma and squamous cell carcinoma, cutan T-cell lymphoma, psoriasis, pityriasis lichenoides chronica or pityriasis rosea [66].

The histological findings also ruled out neoplasia and described a plasma cell infiltrate and endothelial swelling, further non-specific signs of secondary syphilis infection.

Finally, the combination of the dermoscopic image, histological findings and the additionally acquired knowledge of the sexual history of the patient at the second visit led to the diagnosis of secondary syphilis, which was then confirmed with serological tests. Although the nodulo-ulcerative morphology of the lesion raised the possibility of lues maligna, the solitary presentation and the negative HIV test of the patient contradicted this diagnosis.

Dermoscopy may become a supportive tool to facilitate the recognition of secondary syphilis; however, the reporting of these atypical cases is crucial to highlight the many faces of the disease, so that clinicians consider syphilis as part of the differential diagnosis of non-specific lesions.

## 4. Conclusions

Secondary syphilis is well known for physical variability and may present as symmetric macules, nodules or papules, or even as a solitary lesion. It is crucial to highlight the many faces of the disease so that clinicians can consider syphilis in the differential diagnosis of non-specific solitary lesions, especially in high-risk patients.

## Data Availability

Not applicable.
